# Long-Term Effects of Sleeve Gastrectomy on Super Obesity: A Retrospective Cohort Study of Weight Loss and Metabolic Recovery at Centro Médico Nacional 20 de Noviembre

**DOI:** 10.7759/cureus.104581

**Published:** 2026-03-02

**Authors:** Jesús Montoya, Jhon Coronel, Camila Santos, Carlos Acuña, José Rodríguez, Dania Ramírez, Rommel Ramírez, Juan Sánchez

**Affiliations:** 1 General Surgery, Centro Médico Nacional 20 de Noviembre, Mexico City, MEX; 2 General Surgery, Universidad Catolica de Santiago de Guayaquil, Guayaquil, ECU

**Keywords:** bariatric surgery, bmi >50, metabolic disease, sleeve gastrectomy, weight loss

## Abstract

Background: Patients with a body mass index (BMI) >50 kg/m² represent a major clinical challenge because of their high burden of metabolic comorbidities and increased mortality. Laparoscopic sleeve gastrectomy (LSG) is widely used as a primary bariatric procedure; however, evidence regarding its effectiveness in individuals with super obesity remains limited. This study aimed to evaluate weight loss and metabolic outcomes in patients with BMI >50 kg/m² who underwent LSG.

Methods: We conducted an observational, retrospective, longitudinal study at the Centro Médico Nacional 20 de Noviembre, a tertiary care university hospital. Patients with BMI between 50 and 60 kg/m² who underwent primary LSG between January 2020 and December 2024 were included. The maximum institutional follow-up was 24 months. Anthropometric and metabolic variables were analyzed using paired Student’s t test or Wilcoxon test, whereas categorical variables were compared with the chi-square or Fisher’s exact test. Simple linear regression was performed, and statistical significance was set at p < 0.05.

Results: Of 42 patients, 33 met the inclusion criteria. A female predominance was observed (69.7%), and the mean age was 43 ± X years. Mean baseline BMI was 57 ± 4.9 kg/m², decreasing to 35.5 kg/m² at 24 months. Mean weight declined from 150.7 ± 19.9 kg to 96.9 ± 18.7 kg, corresponding to 35.0% total weight loss and 59.6% excess weight loss. Remission rates were 66.7% for type 2 diabetes mellitus, 57.9% for hypertension, and 44.4% for dyslipidemia. HbA1c and HOMA-IR decreased significantly (p < 0.0001). Weight regain and insufficient weight loss occurred in 3.0% of cases each. Complications were infrequent, and no mortality was observed.

Conclusions: LSG in patients with BMI >50 kg/m² achieved substantial and clinically meaningful weight reduction, with most individuals meeting established criteria for surgical success at two years. The procedure was associated with significant improvement and high remission rates of major metabolic comorbidities, while maintaining a favorable safety profile and very low rates of weight regain. These findings support LSG as an effective primary strategy for super-obese patients within a structured multidisciplinary follow-up program. Our results contribute regional evidence that may assist decision-making and counseling in similar high-risk populations.

## Introduction

Extreme obesity, commonly defined as a body mass index (BMI) greater than 50 kg/m², represents a major and growing public health challenge due to its strong association with metabolic comorbidities and increased mortality [1]. Individuals in this category are at markedly higher risk of developing type 2 diabetes mellitus (T2DM), arterial hypertension, dyslipidemia, cardiovascular disease, and reduced quality of life. This burden has driven the search for effective therapeutic strategies capable not only of promoting weight reduction but also of improving the underlying metabolic dysfunction [2].

Bariatric and metabolic surgery has consistently demonstrated superiority over conservative management in achieving sustained weight loss and ameliorating obesity-related comorbidities. Among available techniques, laparoscopic sleeve gastrectomy (LSG) has emerged as one of the most frequently performed procedures worldwide [3]. Originally part of more complex operations such as the duodenal switch, it has progressively evolved into a stand-alone intervention because of its relative technical simplicity, reproducible outcomes, and favorable safety profile [3]. According to a recent report from the International Federation for the Surgery of Obesity and Metabolic Disorders (IFSO), sleeve gastrectomy is currently the predominant primary bariatric procedure in many regions, including the United States [4].

Multiple studies confirm that LSG produces substantial and durable weight loss, with long-term clinical trials reporting an average excess weight loss of 50-60% at five years and maintenance of nearly half that reduction beyond seven to 10 years [5]. Beyond anthropometric improvement, patients frequently experience remission or significant amelioration of diabetes, hypertension, and lipid disorders. Early postoperative morbidity and mortality remain low, with reoperation rates comparing favorably to other bariatric techniques [6].

However, outcomes may vary considerably among patients with more advanced obesity. Individuals with BMI >50 kg/m², often classified as super-obese or super-super-obese, constitute a particularly vulnerable subgroup in whom weight loss may be smaller and persistent comorbidities more frequent [6]. Evidence specific to this population is limited, as many reports combine super-obese patients with lower BMI categories, making it difficult to establish realistic expectations and follow-up strategies.

The assessment of bariatric surgery success has evolved over time. While excess weight loss has traditionally been reported, the percentage of total weight loss (%TWL) is increasingly used because it is less influenced by preoperative BMI and allows more consistent comparisons across populations [6]. International guidelines define therapeutic success as achieving at least 50% excess weight loss or 20% total weight loss, along with meaningful improvement in comorbidities and quality of life [7,8]. Conversely, insufficient weight loss or inability to maintain expected results over time constitutes surgical failure [9,10].

Pharmacological alternatives, such as glucagon-like peptide-1 receptor agonists, have recently expanded obesity management options [11]. While these agents can induce clinically meaningful weight reduction, particularly in mild to moderate obesity, surgical intervention continues to provide the most potent and durable metabolic benefits for patients with severe or extreme BMI elevations [12]. Accordingly, defining the real-world performance of LSG in super-obese patients remains a priority [13,14].

In Latin American populations, particularly in public referral centers, data on medium-term outcomes after sleeve gastrectomy in super-obese patients are scarce [15-17]. Demographic factors, access to multidisciplinary care, and adherence to follow-up protocols may influence surgical results and limit the applicability of international data to local practice.

Therefore, this study aimed to evaluate 24-month weight loss, metabolic comorbidity remission, and safety in patients with BMI >50 kg/m² undergoing primary LSG at a tertiary referral center. By addressing this evidence gap, we sought to clarify the effectiveness and safety of LSG and its impact on obesity-related comorbidities in this high-risk population over a two-year period.

## Materials and methods

This retrospective, observational, longitudinal study was conducted at Centro Médico Nacional 20 de Noviembre between January 2020 and December 2024. No direct intervention was performed, as existing clinical and metabolic data from patients who underwent surgery during this period were analyzed. The study evaluated weight and metabolic changes over time. Although patients operated on within the last five years were included, the institutional follow-up protocol of the Obesity Clinic allows for a maximum follow-up duration of 24 months per patient; therefore, the analysis was limited to this observation period.

Once the project was approved by the research committee of the CMN 20 de Noviembre, the review of patient medical records was conducted. The variables used in the study are shown in Table 1.

**Table 1:** Table of Variables HbA1c: Glycated hemoglobin; HOMA-IR: homeostatic model assessment of insulin resistance

Variable name	Operational definition	Variable type	Unit/scale
Age	Patient age at the time of surgery	Quantitative (continuous)	Years
Sex	Biological sex of the patient	Qualitative (nominal)	Male/Female
Initial weight	Patient's weight before surgery	Quantitative (continuous)	Kilograms (kg)
Height	Patient height	Quantitative (continuous)	Meters (m)
BMI (pre- and postoperative)	Body mass index before surgery and at 1, 3, 6, 12, and 24 months after surgery	Quantitative (continuous)	kg/m²
Postoperative weight	Weight recorded at 1, 3, 6, 12, and 24 months after surgery	Quantitative (continuous)	Kilograms (kg)
%TWL (percent total weight loss)	% of total weight loss at 1, 3, 6, 12, and 24 months relative to baseline weight	Quantitative (continuous)	Percent (%)
%EWL (percent excess weight loss)	% of excess weight loss at 1, 3, 6, 12, and 24 months relative to baseline weight	Quantitative (continuous)	Percent (%)
Baseline HbA1c	Hemoglobin A1c levels before surgery	Quantitative (continuous)	Percent (%)
Postoperative HbA1c	Hemoglobin A1c levels at 24 months after surgery	Quantitative (continuous)	Percent (%)
Baseline HOMA-IR	HOMA-IR index before surgery	Quantitative (continuous)	Index (unitless)
Postoperative HOMA-IR	HOMA-IR index at 24 months after surgery	Quantitative (continuous)	Index (unitless)
Baseline comorbidities	Presence of type 2 diabetes, hypertension, sleep apnea, etc.	Qualitative (nominal)	Yes/No
Diabetes remission	Postoperative diabetes remission (HbA1c < 6.5% without medications), minimum 6 months	Qualitative (dichotomous)	Yes/No
Dyslipidemia remission	Normalization of lipid profile without pharmacologic therapy, minimum 12 months	Qualitative (dichotomous)	Yes/No
Hypertension remission	Normalization of blood pressure without antihypertensive medication, minimum 3 months	Qualitative (dichotomous)	Yes/No
Early and late postoperative complications	Complications occurring ≤30 days or >30 days to 24 months	Qualitative (nominal)	Yes/No
Length of hospital stay	Number of hospitalization days after surgery	Quantitative (discrete)	Days

Definition of comorbidities and clinical outcomes

Preoperative T2DM was defined by a documented medical diagnosis in the clinical record and/or glycated hemoglobin (HbA1c) ≥6.5% prior to surgery, according to established diagnostic criteria [18]. Diabetes remission was evaluated only in patients with a confirmed preoperative diagnosis of T2DM and was defined as HbA1c <6.5% without the use of antidiabetic medication for a minimum period of six months. Arterial hypertension was defined according to institutional diagnostic criteria based on traditional thresholds, as systolic blood pressure ≥140 mmHg and/or diastolic blood pressure ≥90 mmHg on at least two separate clinical visits prior to surgery, or documented use of antihypertensive medication [19]. Hypertension remission was defined as normalization of blood pressure (<140/90 mmHg) without antihypertensive medication for at least three consecutive months during follow-up. Given the severe obesity profile of the cohort (BMI >50 kg/m²), hypertension was classified as obesity-associated essential hypertension. No cases of documented secondary hypertension were identified in the reviewed medical records. 

Postoperative complications were categorized as early (≤30 days) or late (>30 days up to 24 months). Dumping syndrome was not a predefined primary or secondary endpoint but was recorded when clinically documented in the medical records. Diagnosis was based on characteristic postprandial symptoms such as palpitations, diaphoresis, abdominal discomfort, dizziness, or hypoglycemic manifestations after meals. Due to the retrospective design of the study, standardized diagnostic tools (e.g., Sigstad scoring system) were not systematically applied. 

Inclusion criteria

**Figure 1:**
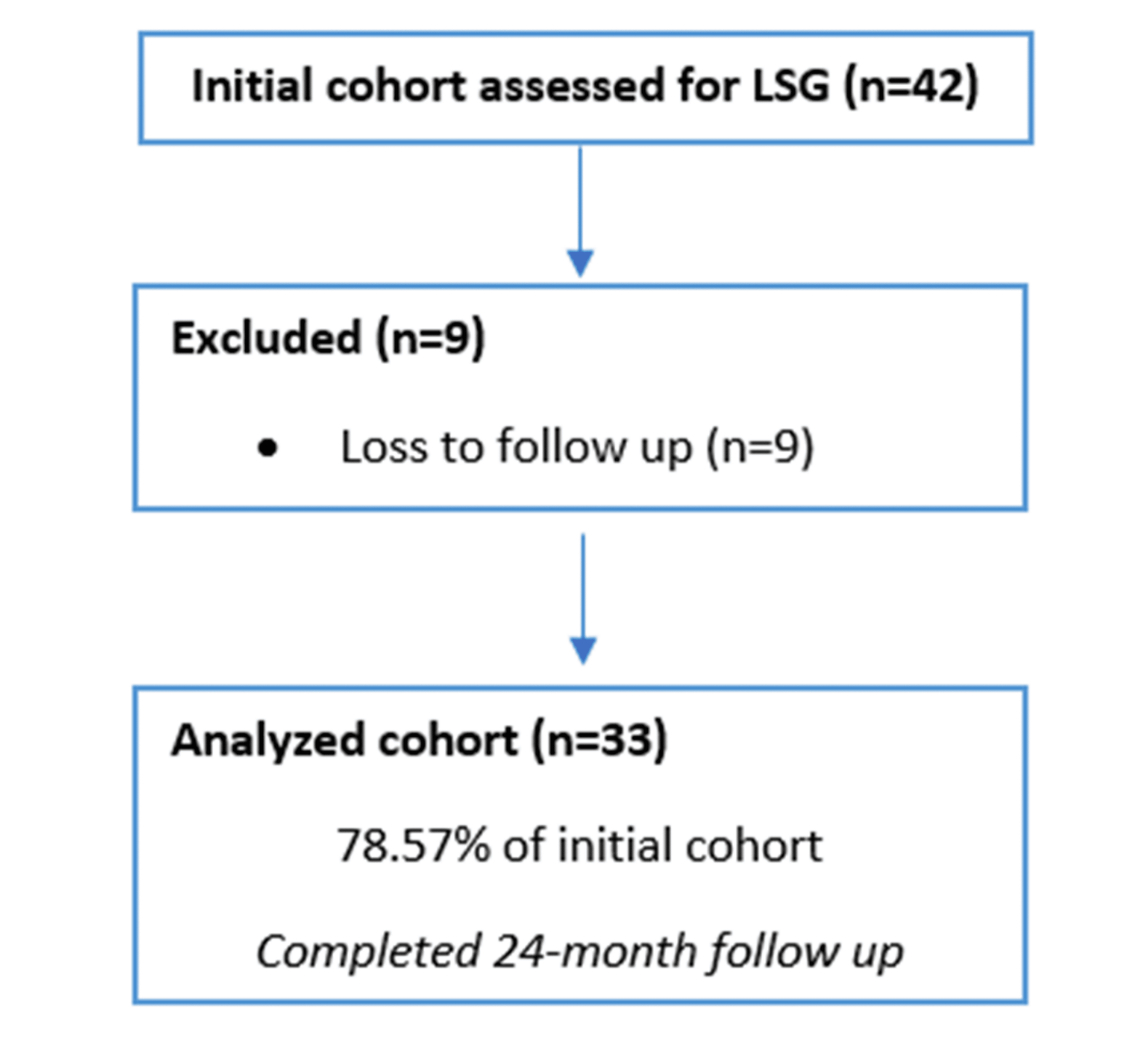
Flowchart of Patient Inclusion Analysis (STROCSS Style) The initial cohort assessed for laparoscopic sleeve gastrectomy (LSG) included 42 patients, of whom nine patients were excluded due to loss to follow-up, leaving a total of 33 patients analyzed for the study with a 24-month follow-up

Eligible participants included patients diagnosed with a BMI >50 kg/m² who underwent primary laparoscopic or robotic sleeve gastrectomy at Centro Médico Nacional 20 de Noviembre. Patients were required to be between 18 and 65 years of age, have at least six months of postoperative follow-up, and have complete clinical data available in their medical records. In accordance with the institutional protocol, the maximum follow-up period considered for analysis was 24 months (Figure 1).

Exclusion criteria

Patients were excluded if they had a BMI >60 kg/m², underwent bariatric procedures other than sleeve gastrectomy, or had previously undergone bariatric surgery (e.g., gastric bypass or biliopancreatic diversion), including revisional procedures. Individuals with severe comorbidities unrelated to obesity that could influence outcomes, such as cancer, severe psychiatric disorders, or advanced chronic degenerative diseases, were also excluded. Additional exclusion criteria included discontinuation of follow-up before six months, pregnancy or breastfeeding, and the presence of genetic syndromes associated with obesity (e.g., Prader-Willi syndrome) (Figure 1).

Elimination criteria

Patients were eliminated from the analysis if they discontinued follow-up before completing six months, developed severe postoperative complications requiring conversion to another bariatric procedure, had incomplete medical records that prevented adequate evaluation of study objectives, or died from causes unrelated to the surgical procedure or medical management (Figure 1).

Sampling methodology

Given the retrospective and observational nature of the study, non-probabilistic convenience sampling was applied. All eligible patients whose medical records were available in the institutional database during the study period were included. No probabilistic or random sampling techniques were used, as the study involved reviewing all accessible cases that met the inclusion criteria.

Sample size determination

The sample size consisted of all patients who fulfilled the inclusion criteria and underwent sleeve gastrectomy within the five-year period prior to this study (2020-2024). Because this was a census-type sample drawn from existing records, no formal sample size calculation was required. Every eligible patient identified in the institutional registry was included.

Statistical analysis

Quantitative variables were summarized using means, standard deviations, and ranges, whereas qualitative variables were expressed as frequencies and proportions. Changes in weight, body mass index, percentage of total weight loss, and percentage of excess weight loss over time were evaluated using linear regression analysis, from which correlation coefficients were obtained. Comparisons between preoperative values and those recorded 24 months after surgery were performed using the paired Student’s t test for normally distributed continuous variables and the Wilcoxon signed-rank test for non-normal distributions. Differences in categorical variables were analyzed with the chi-square test or Fisher’s exact test when expected cell counts were small. A two-tailed p-value < 0.05 was considered statistically significant. Data were collected according to predefined inclusion and exclusion criteria, compiled into a database, and initially structured and cleaned using Microsoft Excel® 2024 (macOS Sonoma v14.7.7, Microsoft Corp., Redmond, WA, USA). Final statistical analyses were conducted with GraphPad Prism® version 10.5.0 (673, GraphPad Software, San Diego, CA, USA), ensuring standardized and reliable data processing.

## Results

During the study period, 42 patients with BMI >50 kg/m² underwent primary LSG; no robotic procedures were performed. Nine patients were excluded due to loss to follow-up, resulting in a final cohort of 33 patients. The cohort was predominantly female (23 women, 69.7%), with a mean age of 43 ± 10.7 years. Preoperative BMI averaged 57 ± 4.9 kg/m² and the mean weight was 150.7 ± 19.9 kg.

**Figure 2:**
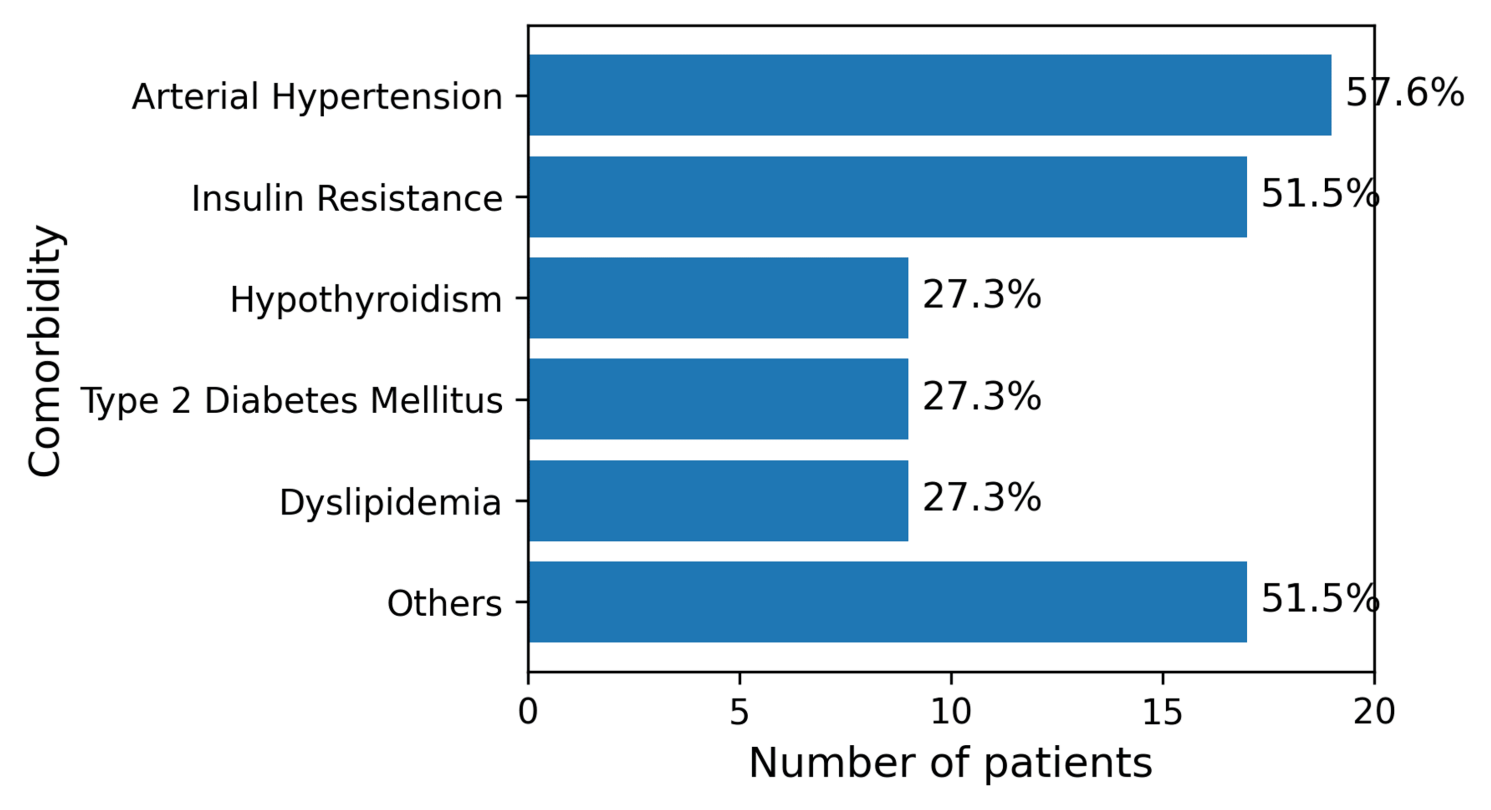
Baseline Comorbidities in Patients with Super Obesity (n =33) Distribution of baseline comorbidities in the study cohort of patients with super obesity (BMI >50 kg/m²) undergoing laparoscopic sleeve gastrectomy (n = 33). Hypertension was the most prevalent comorbidity, followed by insulin resistance. Hypothyroidism, type 2 diabetes mellitus, and dyslipidemia were present in a similar proportion of patients. Other associated conditions included obstructive sleep apnea, osteoarthritis, and additional metabolic disorders.

Baseline comorbidities were common (Figure 2). Arterial hypertension was present in 19 patients (57.6%), insulin resistance in 17 (51.5%), hypothyroidism, T2DM, and dyslipidemia in nine patients each (27.3%), and other conditions, including obstructive sleep apnea, osteoarthritis, or additional metabolic disorders, were identified in 17 patients (51.5%).

In our series, the mean hospital stay was 2.4 ± 1.05 days, reflecting a generally rapid postoperative recovery. As shown in Table 2, no intraoperative complications or mortality were reported. However, two cases of postoperative hemorrhage (6.06%) were identified during early follow-up.

**Table 2:** Intraoperative and Postoperative Data Note: Values are expressed as mean ± standard deviation or as the number of patients (percentage). Complications and mortality refer to the intraoperative or postoperative period. Total number of patients was 33 (n=33)

Intraoperative	
Surgical time (minutes)	96.2 ± 28.3
Complications	0
Mortality	0
Postoperative	
Length of stay (days)	2.4 ± 1.05
Complications	2 (6.06%)
Mortality	0

The first episode occurred in the immediate postoperative period and was resolved with the transfusion of one unit of packed red blood cells, without the need for reoperation. The second episode presented on postoperative day five, prompting hospital readmission and administration of one unit of packed red blood cells; the patient responded favorably to medical management and was discharged after clinical stabilization.

Overall, these findings demonstrate a low complication rate and a short hospital recovery time, with no mortality observed during surgery or in the immediate postoperative period. Also, the findings show a low frequency of complications and a short hospital recovery period, highlighting the absence of mortality both during surgery and in the immediate postoperative phase (Table 2).

**Figure 3:**
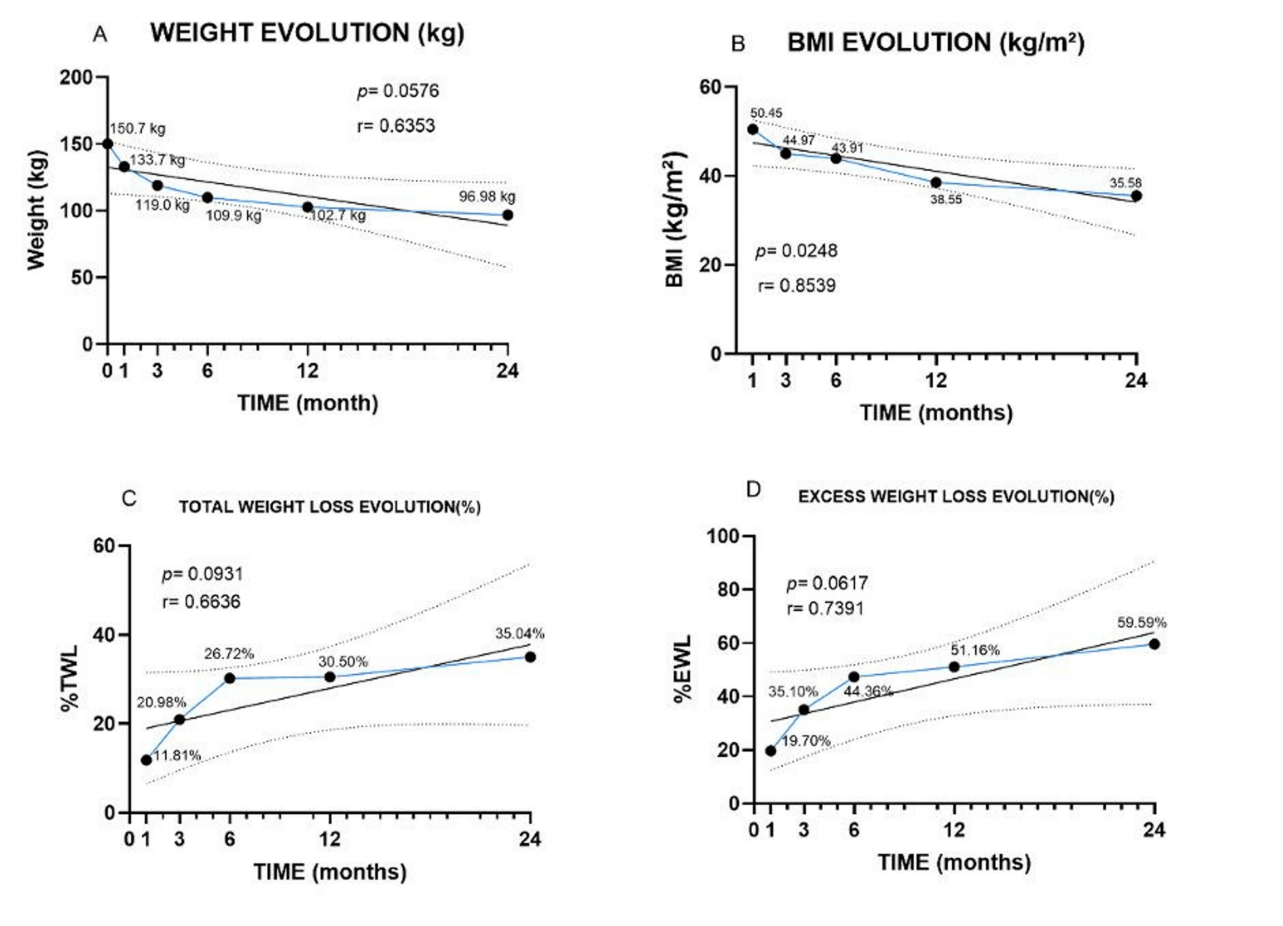
Weight and Anthropometric Parameter Changes Following Laparoscopic Sleeve Gastrectomy Weight and anthropometric parameter changes following laparoscopic sleeve gastrectomy in 33 patients with severe obesity. (A) Weight (kg); (B) BMI (kg/m²); (C) total weight loss (%TWL); (D) excess weight loss (%EWL). Data points represent mean values at each time point (baseline, 1, 3, 6, 12, and 24 months). Error bars indicate standard deviation (SD). Solid lines represent simple linear regression, and dashed lines indicate the 95% confidence interval. p-values correspond to the test for slope ≠ 0; r denotes the correlation coefficient.

Anthropometric outcomes over 24 months are shown in Figure 3. Mean body weight decreased from 150.7 kg to 96.98 kg (p = 0.058; r = 0.635), and BMI from 50.45 to 35.58 kg/m² (p = 0.025; r = 0.854). Percentage total weight loss (%TWL) increased from 11.81% to 35.04% (p = 0.093; r = 0.664), and percentage excess weight loss (%EWL) from 19.70% to 59.59% (p = 0.062; r = 0.739). While not all comparisons reached statistical significance, the trends indicate substantial and clinically meaningful reductions in weight and BMI.

**Figure 4:**
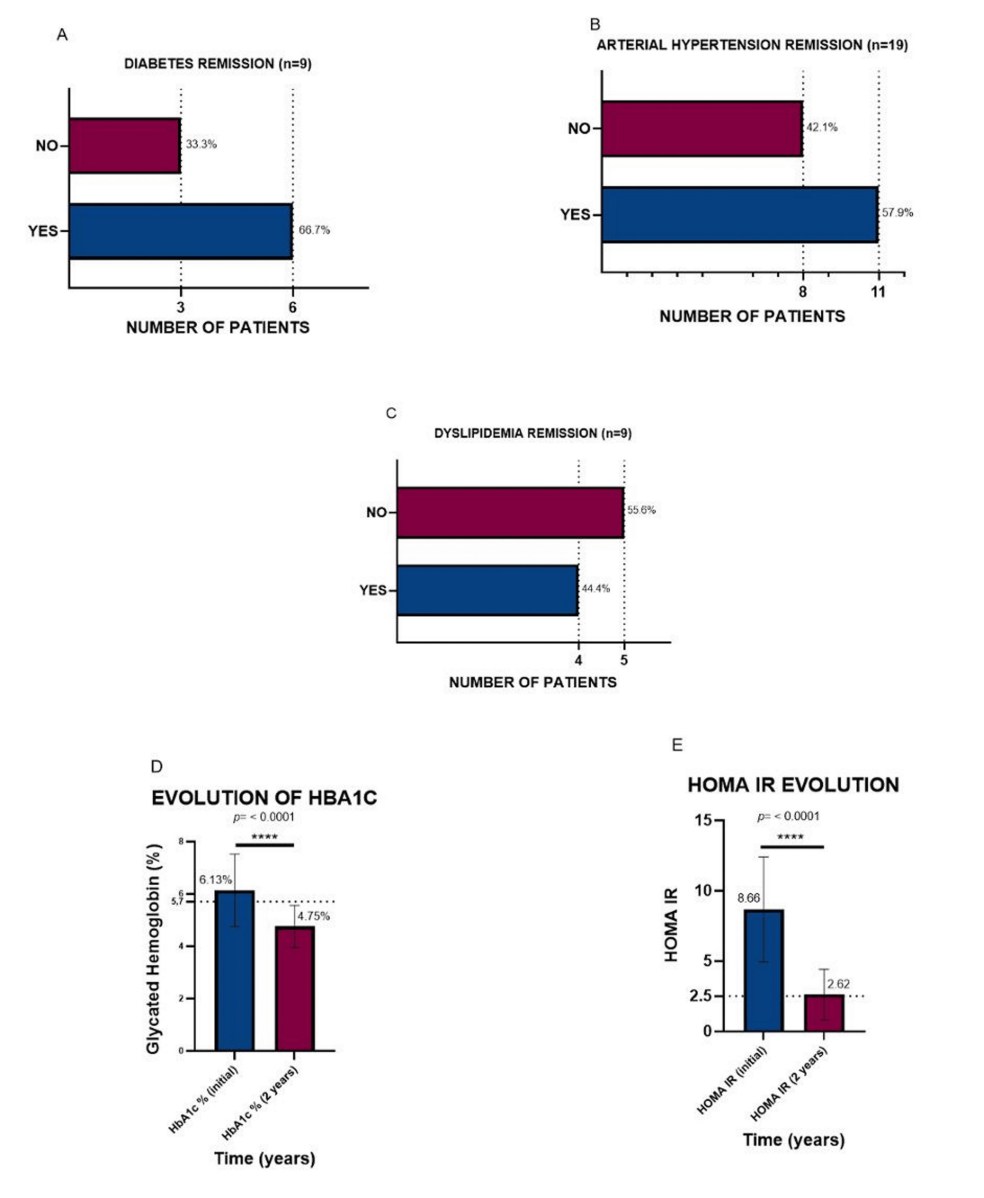
Remission of Comorbidities and Metabolic Parameter Changes in Patients with Severe Obesity Undergoing Laparoscopic Sleeve Gastrectomy (A) Remission of type 2 diabetes mellitus, (B) Remission of arterial hypertension, and (C) Remission of dyslipidemia, all were evaluated at 24 months of follow-up. (D) Glycated hemoglobin (HbA1c) evolution and (E) Homeostatic Model Assessment of Insulin Resistance (HOMA-IR) evolution between baseline and 24 months postoperatively. Data are expressed as the number of patients (percentage) or mean ± standard deviation, as appropriate. Statistical significance is indicated in each panel.

At 24 months (Figure 4), a significant clinical and metabolic impact of sleeve gastrectomy was observed in patients with BMI >50 kg/m². Regarding type 2 diabetes mellitus (Figure 4A), six of nine patients achieved remission criteria (66.7%) without anti diabetic medication, while three patients (33.3%) remained diabetic. Statistical analysis using Fisher’s exact test did not reach significance (p > 0.9999); however, clinically, approximately two out of three patients achieved postoperative glycemic control. For arterial hypertension (Figure 4B), eleven of nineteen patients (57.9%) normalized their blood pressure without pharmacological treatment, whereas eight patients (42.1%) remained hypertensive. Fisher’s exact test again showed no statistical significance (p > 0.9999), but from a clinical perspective, nearly half of hypertensive patients achieved sustained normotension at two years post-surgery. Regarding dyslipidemia (Figure 4C), four of nine patients (44.4%) achieved resolution, while five patients (55.6%) continued to have lipid abnormalities. As with the previous conditions, Fisher’s exact test did not indicate statistical significance (p > 0.9999); nevertheless, clinically, less than half of the patients with dyslipidemia achieved normalization following the procedure. Metabolic biomarkers confirmed these findings. Glycated hemoglobin (HbA1c, Figure 4D) showed a sustained decrease from 6.13% at baseline to 4.75% at two years (p < 0.0001), reflecting consistent improvement in glycemic control. Similarly, the HOMA-IR index (Figure 4E) decreased from 8.66 to 2.62 over the same period (p < 0.0001), approaching reference values compatible with adequate insulin sensitivity.

Overall, these results support the efficacy of sleeve gastrectomy beyond weight loss, demonstrating a profound metabolic impact. High remission rates were observed for diabetes and hypertension, a more modest benefit for dyslipidemia, and sustained biochemical improvements, reinforcing its role as a comprehensive therapeutic strategy in BMI >50 kg/m² gastrectomy.

At the end of the two-year follow-up, only one patient (3.03%) experienced insufficient weight loss, defined as a %TWL below 20% relative to baseline. Similarly, a single case (3.03%) exhibited weight regain, defined as an increase equal to or greater than 10-15% of the weight lost from the nadir.

The low frequency of these events in the cohort suggests that the vast majority of patients maintained an adequate and sustained response to the procedure, achieving clinically meaningful weight loss without evidence of significant regain during the analyzed period.

During follow-up, complications were identified in both the early and late postoperative periods. Within the first 30 days after surgery, two patients (6.06%) developed postoperative bleeding, one occurring in the immediate postoperative period and the other on day five. Both cases were successfully managed with blood transfusion, without progression to major adverse events or the need for reoperation. No anastomotic leaks, thromboembolic events, surgical site infections, or additional early complications were documented.

In the late postoperative period, defined as 30 days to 24 months after surgery, three events were observed. Gastroesophageal reflux disease (GERD) was the most frequent complication, affecting two patients (6.06%), whereas dumping syndrome occurred in one individual (3.03%). No cases of sleeve torsion or angulation, internal hernia, chronic fistula, gastric stenosis, or postprandial hypoglycemia were identified during follow-up.

Overall, the low incidence of adverse events across the study period reinforces the favorable safety profile of LSG in patients with BMI >50 kg/m². Even within this high-risk population, the procedure demonstrated a balanced relationship between efficacy and morbidity, with very few clinically significant complications.

## Discussion

In this cohort of patients with BMI >50 kg/m² undergoing LSG, clinically significant and sustained weight loss was observed at two years of follow-up, with minimal incidence of initial insufficient weight loss (%TWL < 20%) and weight regain ≥10-15% from nadir (3.03% in both cases). This behavior suggests sustained efficacy of the procedure in this high-risk population, consistent with reports by Climent et al. [20], who described substantial weight reduction in patients with BMI >50 kg/m², although some studies indicate a higher risk of long-term weight regain beyond five years of follow-up.

Regarding weight loss efficacy, our findings partially differ from those reported by Cadena-Obando et al. [21], who included 130 patients with severe obesity, 40% of whom were super-obese, and observed that approximately 20% did not achieve ≥50% excess weight loss at one year. In contrast, in our two-year follow-up cohort composed exclusively of patients with super-obese undergoing LSG, the rate of initial insufficient weight loss (%TWL <20%) was much lower (3.03%), with weight regain observed in the same proportion. While Cadena-Obando et al. [21] noted that the type of procedure influences outcomes, with sleeve gastrectomy showing lower success rates, our data demonstrate that under a strict institutional protocol and prolonged multidisciplinary management, this technique can achieve sustained weight loss comparable to malabsorptive procedures. Nevertheless, we concur that high baseline BMI and multiple comorbidities affect both the speed and magnitude of weight loss and may increase long-term failure risk, highlighting the need for structured follow-up and early intervention strategies in patients with suboptimal trajectories.

Metabolic benefits were evident. For T2DM, 66.7% of patients achieved remission at two years, consistent with series reporting resolution rates between 60% and 80% after bariatric surgery, particularly with sustained weight loss. These findings align with ADA guidelines and studies such as that by Schauer et al. [22], which demonstrate that metabolic surgery is more effective than intensive medical therapy for partial or complete remission of type 2 diabetes, especially when weight loss is maintained.

Regarding biomarkers, HbA1c decreased from 6.13% to 4.75%, and HOMA-IR declined from 8.66 to 2.62 over two years (p < 0.0001 for both), confirming sustained improvement in glycemic control and insulin sensitivity. These changes are comparable to those reported by Jiménez et al., who documented reductions greater than 50% in insulin resistance after bariatric surgery, and support the superiority of surgical intervention over intensive medical therapy for metabolic control [23].

In our cohort, arterial hypertension showed a remission rate of 57.9%, consistent with multicenter studies reporting ranges between 43% and 83%. This confirms that sleeve gastrectomy not only promotes weight reduction but also significantly improves cardiovascular comorbidities, even in very high-risk patients.

Dyslipidemia showed a different pattern: only 44.4% of patients achieved normalization, while 55.6% retained lipid abnormalities despite weight reduction. These results contrast with gastric bypass studies, which report higher resolution rates, sometimes approaching 100% in Asian series [24]. A possible explanation for the lower response may relate to the magnitude of weight loss, the procedure type, or baseline lipid profiles. Literature indicates that reductions in triglycerides and LDL are generally proportional to weight loss [25], though the magnitude and speed of improvement vary among patients. In our experience, improvements in glycemic and blood pressure parameters were more consistent and sustained, whereas lipid normalization was more variable [26].

Regarding safety, the incidence of late complications was low: GERD occurred in 6.06% and dumping syndrome in 3.03%, with no perioperative or postoperative mortality. These rates are below those reported in larger series, such as that by Rosenthal et al. [27], where post-sleeve GERD can reach 15-20% and major complications exceed 5%. The absence of severe events, such as stenosis, chronic fistulas, or internal hernias, reinforces the safety of the procedure when performed by experienced teams under structured follow-up protocols.

Finally, the low incidence of initial insufficient weight loss and weight regain in our cohort can be explained by proper patient selection, standardized surgical technique, and multidisciplinary support. Under the Obesity Clinic protocol at Centro Médico Nacional 20 de Noviembre, patients received systematic monitoring over 24 months, allowing reinforcement of lifestyle adherence and early detection of complications or suboptimal weight-loss trajectories. Overall, our results align with international evidence supporting sleeve gastrectomy as an effective and safe option for managing BMI >50 kg/m², with clear benefits in weight reduction, diabetes, and hypertension, although the effect on dyslipidemia is more limited.

Furthermore, recent evidence suggests that adding GLP‑1 agonists preoperatively may enhance surgical outcomes. Liu et al. demonstrated that preoperative semaglutide before sleeve gastrectomy was associated with greater short-term weight loss and higher rates of dyslipidemia remission compared to surgery alone [28].

Despite the strengths of this study, several limitations should be acknowledged. First, the relatively small sample size and single-center design may limit the generalizability of our findings to broader populations. Second, the follow-up period of two years, while sufficient to assess short- and mid-term outcomes, does not allow evaluation of long-term weight maintenance, metabolic stability, or late complications beyond this timeframe. Third, the observational design precludes establishing causal relationships between sleeve gastrectomy and the observed metabolic improvements. Finally, certain variables, such as dietary adherence, physical activity, or psychosocial factors, were not systematically quantified, which could influence individual outcomes. These limitations highlight the need for larger, multicenter, long-term studies incorporating comprehensive lifestyle and behavioral assessments to fully elucidate the efficacy and durability of sleeve gastrectomy in super-obese populations.

## Conclusions

In this cohort of super-obese patients undergoing LSG at Centro Médico Nacional 20 de Noviembre, substantial and sustained weight reduction was observed over 24 months, with minimal incidence of insufficient weight loss or weight regain. Metabolic parameters improved, particularly glycemic control and arterial hypertension, while dyslipidemia resolution was partial, persisting in over half of affected patients. The procedure demonstrated a favorable safety profile, with infrequent late complications and no mortality. These findings indicate that LSG is associated with meaningful weight and metabolic improvements in patients with extreme obesity, particularly when combined with a structured, multidisciplinary follow-up program. Given the retrospective, single-center design and small sample size, these results should be interpreted as observational evidence rather than definitive proof of comparative effectiveness or universal efficacy.
